# Development of a Risk Assessment Model for Early Grade ≥ 3 Infection During the First 3 Months in Patients Newly Diagnosed With Multiple Myeloma Based on a Multicenter, Real-World Analysis in China

**DOI:** 10.3389/fonc.2022.772015

**Published:** 2022-03-17

**Authors:** Yufeng Shang, Weida Wang, Yuxing Liang, Natasha Mupeta Kaweme, Qian Wang, Minghui Liu, Xiaoqin Chen, Zhongjun Xia, Fuling Zhou

**Affiliations:** ^1^ Department of Hematology, Zhongnan Hospital of Wuhan University, Wuhan, China; ^2^ Department of Hematologic Oncology, Sun Yat-sen University Cancer Center, Guangzhou, China; ^3^ Department of Hematologic Oncology, State Key Laboratory of Oncology in South China/Cancer Center, Collaborative Innovation Center for Cancer Medicine, Sun Yat-sen University, Guangzhou, China

**Keywords:** multiple myeloma, infection, risk factors, infection model, novel drug

## Abstract

**Purpose:**

The study aimed to assess factors associated with early infection and identify patients at high risk of developing infection in multiple myeloma.

**Methods:**

The study retrospectively analyzed patients with MM seen at two medical centers between January 2013 and June 2019. One medical center reported 745 cases, of which 540 of the cases were available for analysis and were further subdivided into training cohort and internal validation cohort. 169 cases from the other medical center served as an external validation cohort. The least absolute shrinkage and selection operator (Lasso) regression model was used for data dimension reduction, feature selection, and model building.

**Results:**

Bacteria and the respiratory tract were the most common pathogen and localization of infection, respectively. In the training cohort, PS≥2, HGB<35g/L of the lower limit of normal range, β2MG≥6.0mg/L, and GLB≥2.1 times the upper limit of normal range were identified as factors associated with early grade ≥ 3 infections by Lasso regression. An infection risk model of MM (IRMM) was established to define high-, moderate- and low-risk groups, which showed significantly different rates of infection in the training cohort (46.5% vs. 22.1% vs. 8.8%, *p*<0.0001), internal validation cohort (37.9% vs. 24.1% vs. 13.0%, *p*=0.009) and external validation cohort (40.0% vs. 29.2% vs. 8.5%, *p*=0.0003). IRMM displayed good calibration (*p*<0.05) and discrimination with AUC values of 0.76, 0.67 and 0.71 in the three cohorts, respectively. Furthermore, IRMM still showed good classification ability in immunomodulatory (IMiD) based regimens, proteasome-inhibitors (PI) based regimens and combined IMiD and PI regimens.

**Conclusion:**

In this study, we determined risk factors for early grade ≥ 3 infection and established a predictive model to help clinicians identify MM patients with high-risk infection.

## Introduction

Multiple myeloma (MM) is a malignant disease characterized by the uncontrolled proliferation of clonal plasma cells in bone marrow (1). The overall survival in MM has improved significantly in the past decades with the emergence of thalidomide, bortezomib, lenalidomide, carfilzomib, monoclonal antibodies and hematopoietic stem cell transplantation (2–4). However, these treatment advances have not made a remarkable impact on reducing early mortality (5–7), which cannot be accurately predicted by the presenting prognostic features. The incidence of infection has significantly increased in the recent past and remains a leading cause of death in patients with MM (7). Ample knowledge of the causes of infection comorbidities will raise awareness of early deaths seen in MM and help develop strategies for the early prevention of infection.

Earlier, it was believed that infections in MM were related to deficits in both the humoral and cellular immunity, including B cell dysfunction, numerical and functional abnormalities of the dendritic cells and T cells, and dysfunction of natural killer cells (8–12), as well as poor performance status, which are associated with both the disease and its treatment (13). In a study of 3107 MM patients from the United Kingdom Medical Research Council MM trials, Augustson et al. observed that 45% of early deaths within 6 months were due to infections (14). Moreover, even when early infection was not fatal, it frequently led to substantial delays and dose reduction in subsequent chemotherapy, increasing the risk of treatment failure (15). The indication of antibacterial prophylaxis in patients receiving active MM therapy is controversial. A URCC/ECOG randomized phase III study concluded that the use of prophylactic antibiotics did not decrease the incidence of severe infection or any infection within the first 2 months of treatment (13). Other studies showed that prophylactic use of antibiotics significantly reduced the rate of severe bacterial infection and early infection-related mortality (16, 17). However, prophylactic anti-antibiotics were not recommended for all patients (13, 18, 19). Facon et al. (20) established a model to predict the risk of early grade ≥ 3 infection in patients with MM not eligible for transplant based on clinical trial data. However, only a few studies have addressed the risk factors of early infection in real-world practice.

Identifying patients at high risk of infection in the real world will facilitate individualized treatment options for patients requiring protective strategies. The present study aimed to assess factors associated with early-onset infection in MM and identify patients at high risk of developing infection to determine appropriate infection prevention strategies.

## Patients and Methods

### Patients

**Figure 1:**
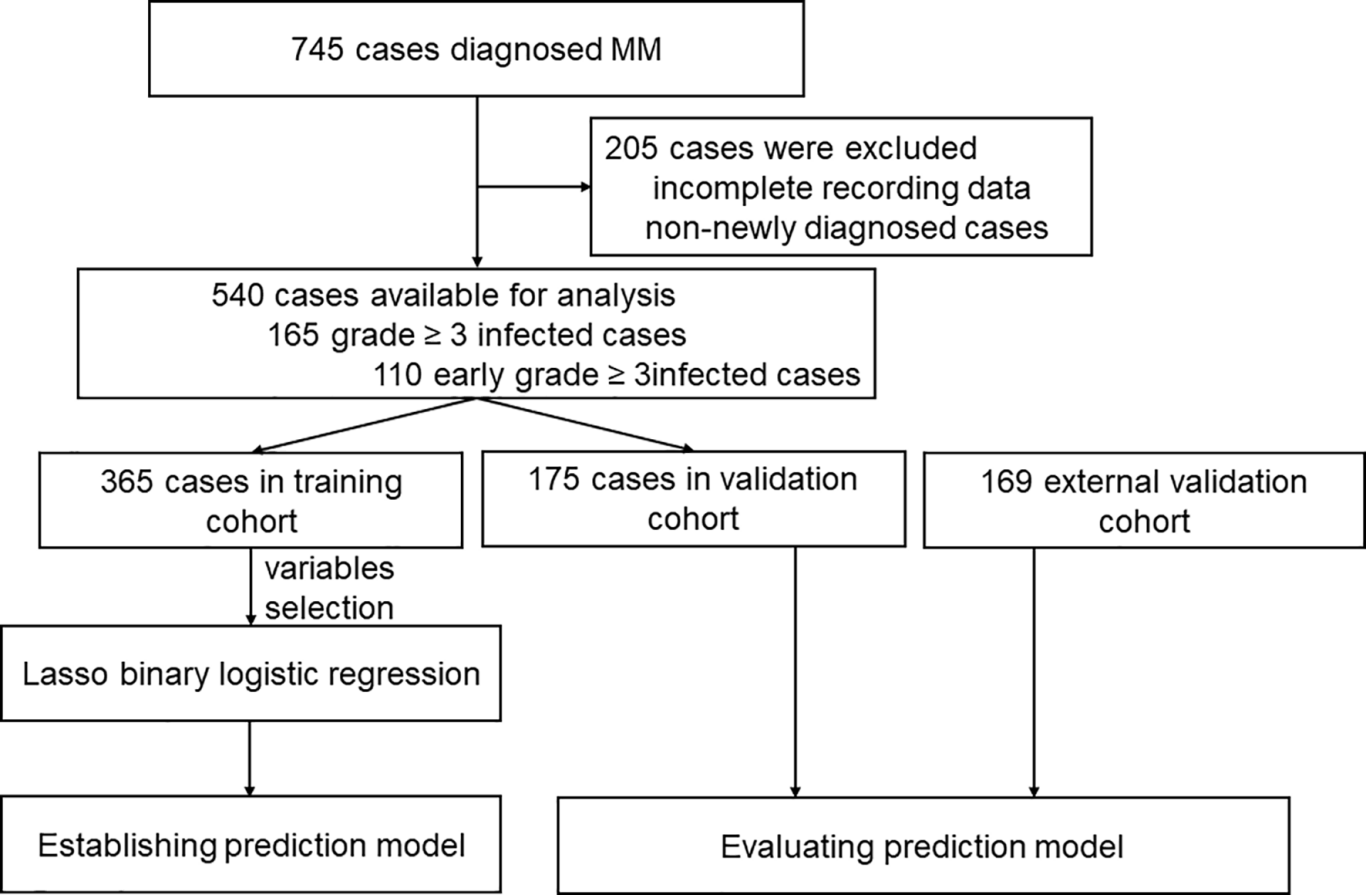
The flow chart of analysis in this study.

Patients diagnosed with MM between January 2013 and June 2019 from Zhongnan Hospital of Wuhan University and Sun Yat-sen University Cancer Center in China were retrospectively analyzed. The flow chart of analysis is illustrated in **Figure 1**. All patients included were diagnosed with MM according to the International Myeloma Working Group (IMWG) criteria (21). Patients with smoldering MM, unclear immunophenotype or solitary plasmacytoma, and patients without adequate clinical information were excluded. The study was approved by the Institutional Review Boards of all participating institutions. All procedures in the study that involved human participants were performed in accordance with the Declaration of Helsinki.

Data were obtained by reviewing medical records. Baseline data collected included general information, including clinical complaints, past medical history, laboratory test results, radiological imaging reports, bone marrow manifestation, immunophenotype, cytogenetics and treatment given. Cytogenetic abnormalities were detected by Fluorescence *in situ* Hybridization (FISH), which were performed on immunologically recognized plasma cells according to the FISH methods of each institution. Imaging examinations included X-ray, computed tomographic (CT) scan imaging, magnetic resonance imaging (MRI) or positron emission tomography-computed tomography (PET-CT) to identify bone destruction or extramedullary lesions. The first-line treatment protocol, including immunomodulatory (IMiD)-based regimens, proteasome-inhibitors (PI)-based regimens and combined IMiD and PI, were recorded in detail. Traditional chemotherapy regimens included MP (melphalan and prednisolone), and VAD (vincristine, adriamycin, dexamethasone), to list a few. The specific regimens received was provided in the Supplementary Appendix.

In addition, the criteria for infection used in this study were identified using patient clinical manifestations, typical imaging findings of infection, or isolation of a microbial agent from peripheral blood or secretions in patients who also had concomitant clinical symptoms. The basic characteristics of identified infections included the type of isolated microbial agents, site of infection, time of occurrence and the duration from the diagnosis of MM to confirmed infection. Antitumor therapy at the time of infection was also ascertained and included. The infection was graded according to Common Terminology Criteria for Adverse Events v3.0 (CTCAE). There is no standard definition of early infection with multiple myeloma. Since the number of patients infected in the first 3 months is the largest and the treatment regimens are relatively uniform, we chose the first 3 months as cut-off to define early infection in this analysis.

### Statistical Analysis

The normality of the distribution of continuous variables was tested by the Kolmogorov-Smirnov test. Patient characteristics were compared using the Chi-square test for categorical variables and t-test or Mann-Whitney U test for continuous variables using IBM SPSS statistics software, version 25.0. The continuous variables with statistical differences were identified as optimum cutoff points using X-tile plots. The least absolute shrinkage and selection operator (LASSO) regression model was performed to screen variables with non-zero coefficients using a package of “glmnet” with R version 3.6.3 software. The coefficients of multivariate analyses were used to generate an infection risk model. To quantify the discrimination performance of the model, the concordance index (C-index) was measured by the bootstrap method with 1000 resampling with the “Hmisc” package. Prognostic accuracy was evaluated by the time-dependent ROC curve analysis with the “survival ROC” package. By comparing observed actual data with the predicted probability of the model, the calibration curves were plotted, accompanied by the Hosmer-Lemeshow test. Decision curve analysis was conducted by quantifying net benefits at different threshold probabilities. Time to first infection was estimated using the Kaplan–Meier method and log-rank test to assess the statistical significance of the difference. All reported *p* values were two-sided at a significance level of 0.05.

## Results

### Characteristics of Patients With Multiple Myeloma

One of the medical centers had 745 reported cases of these 205 patients were excluded due to incomplete data, and additionally, non-newly diagnosed patients previously treated at other hospitals were also excluded. A total number of 540 patients were included for analysis and were divided into training cohort and internal validation cohort (**Figure 1**). Further, 169 patients from the other medical center were included and served as an external validation cohort (**Figure 1**). Baseline characteristics of the 540 newly diagnosed MM in the training and internal validation cohort are shown in **Table 1**. The median age was 59.0 years (range 20.0-85.0). IgG subtype (53.1%) accounted for the largest proportion followed by IgA (21.6%), λ light chain (10.9%), κ light chain (8.5%), Non-secretory (3.0%), IgD (1.5%), IgM (0.7%) and Diclonal (0.7%) subtype. There were no statistical differences in infection among the different subtypes. In total, 165 patients (30.6%) experienced grade ≥ 3 infections during the entire course, while 110 patients (20.4%) experienced an early grade ≥ 3 infection during the first 3 months after diagnosis. Early grade ≥ 3 infection (69.1%) was the highest of all infections reported. In the external validation cohort, 42 of 169 patients (24.9%) suffered from early grade ≥ 3 infection.

**Table 1:** Comparisons of baseline characteristics between infected and uninfected patients in total multiple myeloma cases.

Characteristics	Entire disease course			In the first 3 months		
	Uninfected (n=375) Median (IQR) or No. (%)	Infected (n=165) Median (IQR) or No. (%)	*P*	Uninfected (n=430) Median (IQR) or No. (%)	Infected (n=110) Median (IQR) or No. (%)	*P*
General characteristics						
Age	58.0 (51.0-64.0)	59.0 (53.0-64.5)	0.309	58.0 (51.0-64.0)	60.0 (53.0-66.3)	0.055
Sex/Male	211 (56.3)	109 (66.1)	**0.033**	244 (56.7)	76 (69.1)	**0.019**
ECOG/PS≥2	37 (9.9)	39 (23.6)	**<0.001**	45 (10.5)	31 (28.2)	**<0.001**
Hypertension	52 (13.9)	26 (15.8)	0.565	60 (14.0)	18 (16.4)	0.521
Diabetes	25 (6.7)	10 (6.1)	0.792	27 (6.3)	8 (7.3)	0.706
Cardiac disease	16 (4.3)	13 (7.9)	0.086	18 (4.2)	11 (10.0)	**0.016**
MM subtype			0.212			0.607
IgA	85 (22.7)	33 (20.0)		97 (22.6)	21 (19.1)	
IgG	191 (50.9)	96 (58.2)		223 (51.9)	64 (58.2)	
λ light chain	44 (11.7)	14 (8.5)		48 (11.2)	10 (9.1)	
κ light chain	31 (8.3)	15 (9.1)		35 (8.1)	11 (10.0)	
Non-secretory	14 (3.7)	1 (0.6)		14 (3.3)	1 (0.9)	
^#^Other subtypes	10 (2.7)	6 (3.6)		13 (3.0)	3 (2.7)	
Laboratory findings						
Hemoglobin (g/L)	103.0 (79.0-122.0)	85.0 (71.1-110.0)	**<0.001**	102.3 (79.0-122.0)	81.5 (70.5-101.3)	**<0.001**
WBC (x10^9^/L)	5.9 (4.6-7.3)	5.6 (4.2-7.5)	0.278	5.8 (4.6-7.3)	5.8 (4.3-8.2)	0.951
Platelet (x10^9^/L)	204.7 (149.8-273.5)	178.0 (129.5-242.6)	**0.002**	201.0 (148.0-272.0)	179.0 (137.8-226.0)	**0.007**
Neutrophils (x10^9^/L)	3.3 (2.3-4.6)	3.0 (2.1-4.5)	0.271	3.2 (2.3-4.5)	3.3 (2.1-4.9)	0.709
Lymphocytes (x10^9^/L)	1.8 (1.4-2.3)	1.7 (1.3-2.4)	0.083	1.8 (1.4-2.3)	1.7 (1.3-2.4)	0.503
Monocytes (x10^9^/L)	0.4 (0.3-0.6)	0.4 (0.3-0.7)	0.069	0.4 (0.3-0.6)	0.5 (0.3-0.7)	0.075
Albumin (g/L)	37.1 (31.0-41.8)	32.1 (27.9-39.3)	**<0.001**	36.8 (30.8-41.8)	31.9 (27.2-37.8)	**<0.001**
Globulin (g/L)	46.6 (28.0-71.7)	61.6 (35.0-88.6)	**<0.001**	48.2 (28.9-73.3)	68.4 (34.8-90.6)	**<0.001**
ALP (U/L)	75.1 (59.3-98.4)	73.5 (55.8-97.1)	0.340	75.9 (59.4-98.3)	69.9 (51.5-96.8)	0.132
Ca (mmol/L)	2.3 (2.2-2.4)	2.3 (2.1-2.5)	0.984	2.3 (2.2-2.4)	2.3 (2.0-2.5)	0.725
Creatinine (umol/L)	77.7 (58.9-105.1)	87.7 (67.8-155.9)	**<0.001**	78.8 (59.1-104.8)	95.2 (72.0-174.3)	**<0.001**
CRP (mg/L)	3.0 (1.0-9.8)	4.2 (1.2-11.6)	0.162	3.0 (1.0-9.4)	5.6 (1.2-13.6)	**0.039**
LDH (U/L)	166.0 (133.6-210.2)	166.6 (133.7-223.6)	0.456	165.3 (133.8-209.0)	175.0 (132.7-241.7)	0.133
Uric acid (umol/L)	417.9 (325.0-512.8)	457.8 (357.7-571.4)	**0.004**	418.5 (326.1-515.7)	467.8 (359.9-587.3)	**0.003**
β2-MG (mg/L)	4.3 (2.9-6.6)	6.2 (3.7-10.3)	**<0.001**	4.3 (2.9-6.6)	6.9 (4.5-12.3)	**<0.001**
M protein (%)	31.5 (14.8-46.3)	42.0 (23.3-54.7)	**0.001**	32.4 (15.3-46.8)	43.4 (28.9-54.7)	**0.001**
Plasm cell in BM (%)	15.0 (6.0-32.0)	19.0 (7.5-38.3)	**0.044**	15.0 (6.0-32.0)	21.5 (8.3-42.6)	**0.010**
Bone lesions >3	255 (68.0)	133 (80.6)	**0.003**	301 (70.0)	87 (79.1)	0.059
Renal dysfunction	57 (15.2)	39 (23.6)	**0.018**	66 (15.3)	30 (27.3)	**0.004**
Stage						
ISS I	104 (27.7)	26 (15.8)	**<0.001**	118 (27.2)	12 (10.9)	**<0.001**
II	142 (37.9)	47 (28.5)		162 (37.7)	27 (24.5)	
III	129 (34.4)	92 (55.8)		150 (34.9)	71 (64.5)	
RISS I	61 (20.0)	10 (8.1)	**<0.001**	65 (19.0)	6 (7.0)	**<0.001**
II	208 (68.2)	88 (71.0)		238 (69.4)	58 (67.4)	
III	36 (11.8)	26 (21.0)		40 (11.7)	22 (25.6)	
DS I	20 (5.3)	3 (1.8)	**<0.001**	21 (4.9)	2 (1.8)	**0.004**
II	56 (14.9)	9 (5.5)		59 (13.7)	6 (5.5)	
III	299 (79.7)	153 (92.7)		350 (81.4)	102 (92.7)	
Cytogenetic Abnormalities						
RB1	38 (17.4)	17 (18.1)	0.876	43 (17.3)	12 (18.8)	0.781
1q21	50 (23.7)	28 (30.8)	0.198	59 (24.5)	19 (31.1)	0.288
p53	17 (7.9)	7 (7.5)	0.909	19 (7.8)	5 (7.9)	0.962
D13S319	28 (13.3)	21 (23.3)	**0.030**	36 (14.9)	13 (21.7)	0.206
IGH	29 (13.4)	24 (25.5)	**0.009**	40 (16.3)	13 (20.3)	0.443
High risk CA	28 (12.5)	15 (15.3)	0.496	35 (13.8)	8 (11.8)	0.664
Treatment			0.930			0.158
IMiD-based	52 (66.7)	26 (33.3)		66 (84.6)	12 (15.4)	
PI-based	139 (68.5)	64 (31.5)		154 (75.9)	49 (24.1)	
Combined IMiD and PI	74 (66.7)	37 (33.3)		92 (82.9)	19 (17.1)	

Significant P values are in bold.

P values were calculated by Mann-Whitney U test, χ² test, or Fisher’s exact test, where appropriate.

^#^Other subtypes included 4 cases of IgM, 8 cases of IgD, and 4 cases of Diclonal MM.

β2-MG, β2-microglobulin; ALP, alkaline phosphatase; CA, Cytogenetic Abnormalities; Chemo, Traditional chemotherapy; COPD, chronic obstructive pulmonary disease; CRP, C reactive protein; DS, Durie-Salmon; ECOG PS, Eastern Cooperative Oncology Group performance status; IMiD, immunomodulatory drugs; ISS, International Staging System; LDH, lactate dehydrogenase; PI, proteasome inhibitors; R-ISS, Revised-ISS; VRD, bortezomib, lenalidomide, dexamethasone.

### Characteristics of Early Grade ≥ 3 Infection in the First 3 Months After Diagnosis

**Figure 2:**
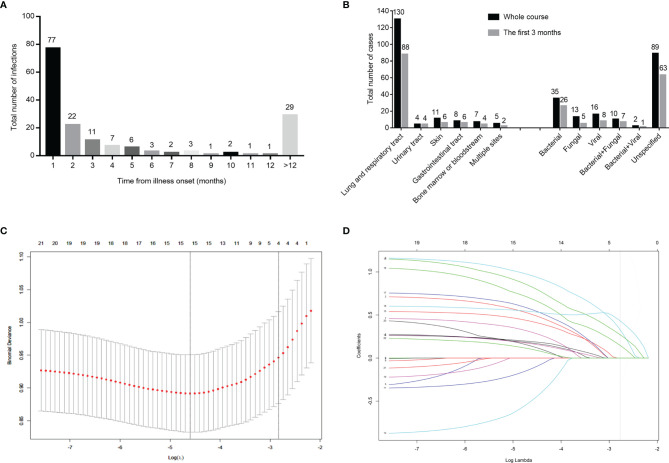
Distribution of infection among540 patients in the training cohort and internal validation cohort, and clinical feature screening using LASSO binary logistic regression model in the training cohort. Number of infected cases monthly in 540 newly diagnosed multiple myeloma patients **(A)**. Number of patients with infections by the site of infection and species of pathogens **(B)**. Four variables selected by LASSO binary logistic regression analysis. Two dotted vertical lines indicate the optimal values by minimum criteria and 1-s.e. criteria **(C)**. LASSO coefficient profiles of the 24 variables. The vertical line indicates the optimal value based on the 1-s.e. criterion giving four non-zero coefficients **(D)**. LASSO, the Least Absolute Shrinkage and Selection Operator.

Of the 110 early grade ≥ 3 infections in the training cohort and internal validation cohort, 49 patients were treated with a PI-based regimen at the time of infection, 12 patients were treated with an IMiD-based regimen, whereas 19 patients were treated with a combined IMiD and PI regimen. The above treatment regimens showed no statistical difference to the occurring infection. The number of infections per month was highest during the first 3 months of diagnosis with 77 cases reported in the first month, 22 cases in the second month, and 11 cases in the third month, respectively (**Figure 2A**). The lungs and respiratory tract were sites involved in 80.0% of early infections, the skin and gastrointestinal tract were sites involved in 5.5% of cases, respectively, and the urinary tract was involved in 3.6% of cases. Similarly, the bone marrow and bloodstream were other sites of involvement (**Figure 2B**). Pathogens were identified in 42.7% of early infections from secretions or peripheral blood; bacterial infections were implicated in 53.3% of the cases followed by viral infections (17.0%) and fungal infections (10.6%) (**Figure 2B**). Lastly, 8 patients were infected with multiple microbiology. The specific types of the microbiology of infections are shown in **Supplementary Table S1**. The localization and microbiology of infections by treatment arm during the first 3 months and the whole course are shown in **Supplementary Table S2**.

### Comparison of Baseline Variables Between Infected and Non-Infected Groups

As shown in **Table 1**, male gender, poor performance status (PS), advanced disease stage, renal dysfunction and multiple bone lesions had a higher incidence of infection (*p*<0.05). Hemoglobin (HGB) level, platelet count, serum albumin, globulin, creatinine, uric acid and β2-microglobulin (Sβ2M) showed statistical differences between the infected and non-infected groups. M protein level and bone marrow plasma cells also showed statistical differences between the two groups. Cytogenetic abnormalities showed no statistically significant differences between the infected and non-infected groups in the first 3 months after diagnosis (*p*>0.05).

### Clinical Characteristics in the Training Cohort, Internal Validation Cohort and External Validation Cohort

Data of the 540 patients were grouped into the training and internal validation cohort by the random digital grouping method. Demographic and baseline characteristics of the training and internal validation cohort are summarized in **Table 2**. Of the 365 patients in the training cohort, 94 patients had infection, and of the 175 patients in the internal validation cohort,36 patients were reported to have infection. No significant differences were observed in clinical characteristics between the training and internal validation cohorts (*p*>0.05), which justified their use as training and internal validation cohorts (**Supplementary Table S3**). Additionally, of the 169 cases in the external validation cohort, 42 had early grade ≥3 infections. The comparison between infected and non-infected patients during the first 3 months in the training cohort, internal validation cohort and external validation cohort are shown in **Table 2**.

**Table 2:** Distribution and comparisons of infected patients and uninfected patients in the first 3 months in the training cohort, internal validation cohort and external validation cohort.

Characteristics	Training cohort (n=365)			Internal validation cohort (n=175)			External validation cohort (n=169)		
	Uninfected (n=291) Median (IQR) or No. (%)	Infected (n=74) Median (IQR) or No. (%)	*P*	Uninfected (n=139) Median (IQR) or No. (%)	Infected (n=36) Median (IQR) or No. (%)	*P*	Uninfected (n=127) Median (IQR) or No. (%)	Infected (n=42) Median (IQR) or No. (%)	*P*
Sex/Male	170 (58.4)	55 (74.3)	0.012	74 (53.2)	21 (58.3)	0.584	74 (58.3)	25 (59.5)	0.886
Age (years)	57.0 (51.0-64.0)	60.0 (50.8-68.0)	0.169	59.0 (51.0-64.0)	60.0 (56.3-64.8)	0.186	63.0 (56.0-70.0)	65.5 (58.8-72.3)	0.089
>65 years	53 (18.2)	23 (31.1)	0.015	27 (19.4)	7 (19.4)	0.998	52 (40.9)	21 (50.0)	0.304
ECOG PS≥2	30 (10.3)	22 (29.7)	<0.001	15 (10.8)	9 (25.0)	0.027	16 (12.6)	15 (35.7)	0.001
HGB (g/L)	102.0 (79.0-123.0)	80.0 (67.0-98.3)	<0.001	104.0 (79.0-120.0)	86.0 (74.6-110.0)	0.020	86.4 (70.1-105.0)	71.3 (58.5-91.4)	0.005
Anemia	120 (41.2)	55 (74.3)	<0.001	55 (39.6)	21 (58.3)	0.043	63 (49.6)	29 (69.0)	0.028
PLT (x10^9^/L)	198.0 (148.0-269.0)	174.5 (129.8-233.5)	0.033	206.0 (147.0-286.0)	185.0 (153.3-239.5)	0.154	160.5 (107.8-203.3)	159.0 (112.8-217.0)	0.739
<150 x10^9^/L	75 (25.8)	25 (33.8)	0.168	36 (25.9)	9 (25.0)	0.912	58 (46.0)	18 (42.9)	0.720
ALB (g/L)	36.7 (30.7-41.8)	31.7 (27.0-38.05)	<0.001	36.7 (30.9-41.6)	32.3 (27.8-37.7)	0.025	33.3 (27.3-37.1)	29.4 (21.8-33.0)	0.004
<30g/L	65 (22.3)	26 (35.1)	0.023	27 (19.4)	12 (33.3)	0.074	44 (34.6)	22 (53.7)	0.030
GLB (g/L)	49.0 (28.9-73.7)	79.0 (33.2-90.6)	0.002	46.8 (29.1-74.6)	60.7 (36.4-93.7)	0.034	49.3 (28.1-69.1)	65.9 (38.7-81.1)	0.022
≥2.1fold	69 (23.7)	37 (50.0)	<0.001	35 (25.2)	16 (44.4)	0.023	41 (32.3)	23 (56.1)	0.006
CREA (umol/L)	78.6 (60.2-102.1)	101.2 (72.8-191.0)	<0.001	79.7 (58.4-108.0)	91.7 (58.3-143.9)	0.171	92.8 (67.0-159.0)	117.5 (73.7-377.3)	0.051
≥110umol/L	63 (21.6)	35 (47.3)	<0.001	34 (24.5)	14 (38.9)	0.084	47 (37.0)	21 (50.0)	0.137
UA (umol/L)	417.1 (325.2-527.4)	477.6 (374.9-627.1)	0.001	423.4 (330.4-504.7)	450.7 (336.2-511.2)	0.631	374.9 (282.9-490.8)	499.0 (379.8-630.6)	<0.001
>620umol/L	27 (9.3)	20 (27.0)	<0.001	8 (5.8)	3 (8.3)	0.570	16 (12.7)	13 (31.0)	0.007
LDH (U/L)	164.7 (134.5-206.6)	182.3 (137.2-257.6)	0.111	168.3 (130.1-211.5)	169.9 (129.0-237.2)	0.734	168.0 (129.5-222.5)	184.0 (129.5-310.0)	0.341
≥215U/L	64 (22.0)	28 (37.8)	0.005	31 (23.0)	11 (30.6)	0.349	32 (29.4)	13 (36.1)	0.448
β2-MG (mg/L)	4.4 (2.8-6.8)	6.9 (4.8-12.9)	<0.001	4.2 (3.0-6.4)	6.8 (3.3-10.1)	0.007	5.6 (3.1-10.1)	10.4 (5.2-17.5)	0.001
≥6 mg/L	87 (29.9)	47 (63.5)	<0.001	42 (30.2)	20 (55.6)	0.005	60 (48.4)	29 (72.5)	0.008
CRP (mg/L)	2.9 (1.1-9.3)	6.0 (1.2-15.3)	0.057	3.2 (0.8-9.9)	5.1 (1.6-15.9)	0.166	4.4 (1.4-12.2)	17.7 (7.4-53.3)	<0.001
>8.5mg/L	77 (26.5)	34 (45.9)	0.001	41 (29.5)	12 (33.3)	0.655	19 (20.0)	11 (50.0)	0.004
M protein (%)	28.9 (12.0-44.4)	38.2 (15.4-53.7)	0.004	24.5 (10.5-44.0)	39.7 (18.8-53.1)	0.034	–	–	–
>50%	48 (16.5)	24 (32.4)	0.002	25 (18.0)	12 (33.3)	0.044	–	–	–
BMPC%	15.0 (6.5-32.4)	16.3 (6.5-37.0)	0.587	15.5 (5.0-31.0)	31.5 (15.3-51.2)	<0.001	23.3 (15.5-36.3)	24.5 (15.6-39.8)	0.419
>50%	30 (10.3)	11 (14.9)	0.268	11 (7.9)	10 (27.8)	0.003	11 (11.0)	7 (21.2)	0.150
Bone lesions >3	210 (72.2)	60 (81.1)	0.119	91 (65.5)	27 (75.0)	0.277	72 (57.1)	25 (59.5)	0.787
ISS III	103 (35.4)	51 (68.9)	<0.001	47 (33.8)	20 (55.6)	0.023	63 (50.4)	28 (71.8)	0.019
DS III	243 (83.5)	69 (93.2)	0.034	107 (77.0)	33 (91.7)	0.050	98 (77.8)	37 (88.1)	0.145
Renal dysfunction	43 (14.8)	22 (29.7)	0.003	23 (16.5)	8 (22.2)	0.427	44 (34.6)	14 (33.3)	0.877
Cardiac disease	13 (4.5)	7 (9.5)	0.146	5 (3.6)	4 (11.1)	0.088	20 (15.7)	11 (26.2)	0.130
Hypertension	39 (13.4)	12 (16.2)	0.533	21 (15.1)	6 (16.7)	0.818	45 (35.4)	18 (42.9)	0.388
Diabetes	17 (5.8)	4 (5.4)	0.999	10 (7.2)	4 (11.1)	0.490	20 (15.7)	6 (14.3)	0.820

P values were calculated by the Mann-Whitney U test, χ² test, or Fisher’s exact test, when appropriate.

ALB, albumin; β2-MG, β2-microglobulin; BM, bone marrow; CREA, Creatinine; CRP, C reactive protein; DS, Durie-Salmon; ECOG PS, Eastern Cooperative Oncology Group performance status; GLB, globulin; HGB, hemoglobin; ISS, International Staging System; LDH, lactate dehydrogenase. BMPC, Bone Marrow Plasma cell; PLT, platelet; UA, uric acid.

### Building the Infection Risk Model of Multiple Myeloma

Based on the variables that were statistically significant in the infected and non-infected groups, and the variables with clinical significance, a total of 24 variables were analyzed by LASSO binary logistic regression. The 4 variables that were reliably associated with early infection were then screened in the training cohort (**Figures 2C, D**). The weight for each factor associated with infection was obtained by calculating the coefficients when log(λ)=-2.83 and λ=0.0588 from the Lasso binary logistic regression model (**Figures 2C, D**). The coefficients for each parameter were as follows: 0.3761 for PS≥2, 0.2950 for HGB<35g/dL of the lower limit of normal range, 0.4581 for β2MG>6.0mg/L, and 0.1706 for GLB≥2.1 times the upper limit of normal range. According to multivariate analysis and coefficients of Lasso binary logistic regression, an early Infection Risk model of Multiple Myeloma (IRMM) was generated (**Table 3**). Based on the best sensitivity/specificity ratio, a cutoff value was selected. We defined 0 to 1 point as low risk, 2-3 points as moderate risk, and 4-6 points as high risk.

**Table 3:** The parameters and groupings of our IRMM.

Variables	Our Points	Infection Risk
ECOG PS of ≥ 2	2	*Low risk, 0-1 points* *Moderate risk, 2-3 points* *High risk, 4-6 points*
Sβ2M ≥ 6 mg/L	2	
HGB < 35g/L of the lower limit of normal range	1	
GLB ≥ 2.1times the upper limit of normal range	1	

β2-MG, β2-microglobulin; ECOG PS, Eastern Cooperative Oncology Group performance status; GLB, globulin; HGB, hemoglobin.

### Performance of the IRMM in the Training and Independent Validation Cohort

**Figure 3:**
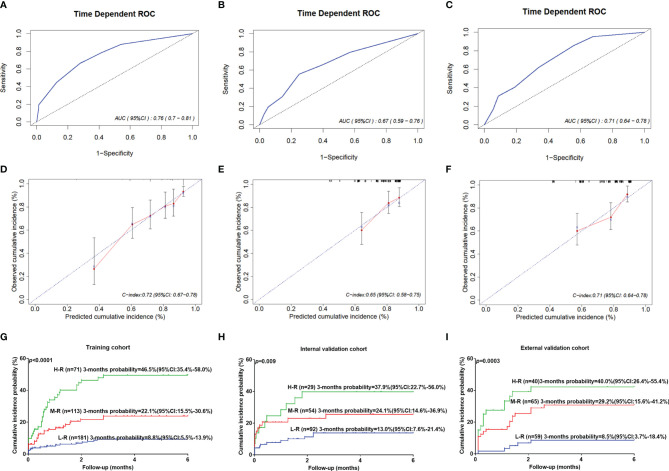
The performance of IRMM to predict early grade ≥3 infection during the first 3 months of MM diagnosis. Time-dependent ROC curves and AUCs at 3 months to assess the prediction accuracy of IRMM in the training cohort **(A)**, internal validation cohort **(B)**, and external validation cohort **(C)**. Calibration curves of the IRMM in the training cohort **(D)**, internal validation cohort **(E)**, and external validation cohort **(F)**. Time to infection in the first 3 months for high-, moderate- and low-risk groups in the training cohort **(G)**, internal validation cohort **(H)**, and external validation cohort **(I)**. AUC, area under the curve; H-R, High risk; L-R, Low risk; M-R, Moderate risk; ROC, receiver operating characteristic.

The model showed great prognostic accuracy in the training cohort, internal validation cohort and external validation cohort by time-dependent ROC analysis (**Figures 3A–C**). The performance of this model was validated by calibration plots for the probability of infection, which demonstrated good agreement between IRMM prediction and the actual observation in the training cohort (**Figures 3D–F**). The Hosmer-Lemeshow test yielded a nonsignificant statistical relationship (*p*<0.05), indicating that there was no departure from a perfect fit. C-index of IRMM was 0.72 (95% CI, 0.67 to 0.78) for training cohort, 0.65 (95% CI, 0.58 to 0.75) for internal validation cohort, and 0.71 (95% CI, 0.64 to 0.788) for external validation cohort. For illustration, a time-to-first infection analysis was performed in the three cohorts. The results concluded that patients in the high-risk group had the highest probability of early grade ≥ 3 infection in the first 3 months after diagnosis compared with patients in the moderate-risk and low-risk groups (**Figures 3G–I**).

### Clinical Use and the Performance of IRMM in the Different Treatment Regimens

**Figure 4:**
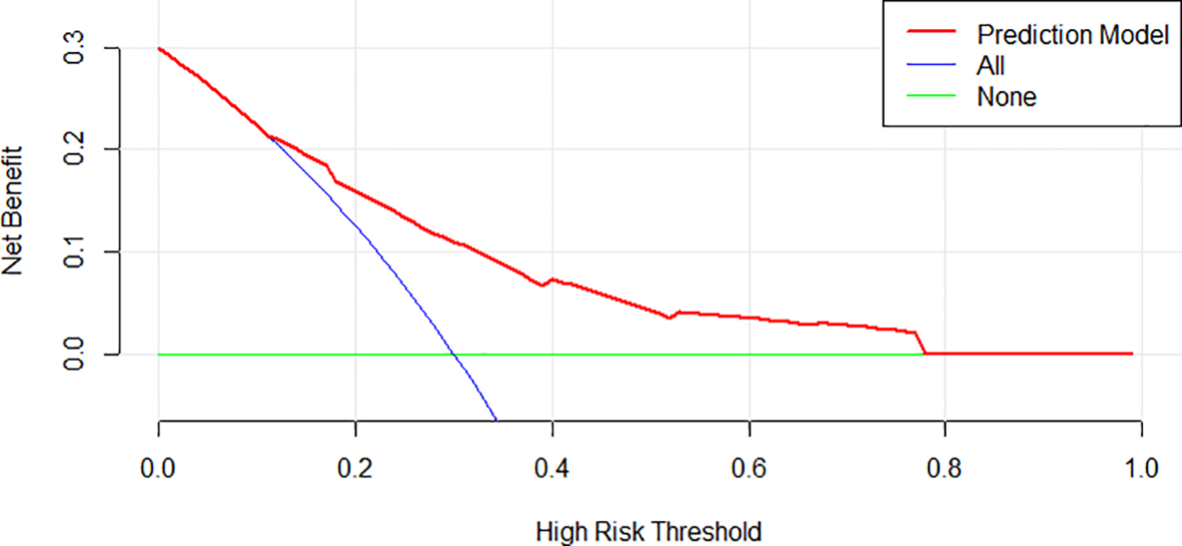
Decision curve analysis for IRMM. The y-axis measures net benefit. The red line represents IRMM. The green line represents the assumption that all patients have an infection.

The decision curve analysis for IRMM is demonstrated in **Figure 4**. It showed that if the threshold probability of a patient or doctor is 10%, using IRMM to predict infection added more benefits than the treat-all-patients scheme or the treat-none scheme.

To understand the distinguishing ability of IRMM in the PI-based regimen, IMiD-based regimen and combined IMiD and PI regimen, we further compared the infection possibilities of the three risk groups in different treatment regimens. In the training cohort and external validation cohort, patients in the high-risk group had the highest probability of early grade ≥ 3 infection than patients in the moderate-risk and low-risk group based on the three treatment regimens (**Supplementary Figure S1**).

## Discussion

This analysis showed that two-thirds of patients experience a first episode of infection in the first 3 months of diagnosis. Our results are consistent with the findings of previous studies that concluded that infections commonly occur in the first and second months of treatment (20). Infections remain a significant cause of morbidity and mortality in patients with MM (11). Early deaths mainly due to associated comorbidities occur before the maximum beneficial effect of chemotherapy in reducing tumor load is achieved. The risk of infection in the first 6 months after diagnosis ranges from approximately 20% to 55%. 20% of deaths reported within 2 months are attributed to infection (15, 22). As shown in previous similar studies, upper airway and lung infections were the most common, affecting the higher proportion of patients (14, 20). One of the reasons might be because many patients experienced bone pain, which impaired normal ventilation, reduced the clearance of secretions, and often required narcotic analgesics that might suppress ventilation and promote or lead to infections of the upper airway and lungs. The percentage of early grade ≥3 infections is higher compared to other reports (20). In addition to the prevention of herpes virus infection, we do not routinely perform other positive antibacterial prophylaxis for patients, so that may be one of the reasons for the high rate of infection. It makes sense to develop infection risk models in the absence of preventive anti-infection interventions. This scoring model can remind clinicians to the use of prophylactic antibiotics in high-risk patients.

In patients with infections and pathogenic results, 72.3% of patients had bacterial infections, 25.5% had fungal infections, and 19.1% had viral infections during the first 3 months, similar to the reported rates of bacterial infections of around 50% to 90% (7, 19, 23, 24). The incidence of fungal infection was slightly higher in the present analysis, which may be due to various unspecified pathogens. More than half of cases with fungal infections were accompanied by bacterial infections. The rate of viral infections was similar to the Swedish study (7). In cases reported with viral infections, all the patients were treated with bortezomib-based regimens, including 7 patients with Herpes zoster and 2 patients with viral influenza, without hepatitis B virus (HBV) and cytomegalovirus (CMV) reactivation. A Japanese study reported that HBV reactivation occurred in 7.7% of patients, with a cumulative incidence at 2 and 5 years (8% and 14%), respectively (25). The risk of HBV and CMV reactivation mainly occurred after autologous stem cell transplant (ASCT) (25, 26). We speculate that the probability of virus reactivation in the first three months of diagnosis was relatively low, but the risk of virus reactivation in the advanced stage was exigent.

In this retrospective analysis, we mainly studied infection incidences in the first three months of diagnosis to avoid confounding factors and censored data resulting from long-term follow-up. Individual patient factors, inherent tumor characteristics and clinical indicators were included to explore the risk factors of infection. For different treatment regimens, the risk of early infection was similar (*p*=0.158) across various treatment regimens, highlighting the role of baseline patient-specific factors in determining infection risk and the complexity of the innate and specific immunosuppression (27). PS, anemia, elevated β2MG and GLB were identified as factors associated with early infection. IRMM was established to categorize patients into high- moderate- and low-risk groups, which showed significantly different rates for early infection in the three cohorts. This strategy has a significant clinical implication for guiding clinicians on the application of prophylactic antibiotics in various populations to avoid the overuse of antibiotics. It is imperative because there is insufficient data and unclear guidelines regarding which prophylactic antibiotic is ideal for specific patient populations, and determining the indication of anti-infectious prophylaxis in patients receiving active MM therapy is controversial (19).

IRMM demonstrated good predictive performance and clinical net benefit. Our conclusions were derived from real-world research, avoiding strict control from clinical trials. Similar to the Facon study (20), PS, HGB and serum β2MG were associated with infection. Elevated serum GLB, which is one of the features of MM and partially indicates a high tumor burden, was included in the model for the first time to predict infection (20, 28). Previously, immunoparesis and polyclonal hypogammaglobulinemia in MM patients was regarded as an important risk factor for infection with encapsulated bacteria (29), such as Streptococcus pneumoniae (30) and Haemophilus influenzae (31). Elevated serum GLB may partially reflect an increase in non-functional monoclonal immunoglobulins that lead to immunoparesis. For this reason, GLB was included in the infection risk model.

Our study had some limitations. Firstly, infection types predicted by our model included bacteria, fungi, viruses, *etc.*, without distinct classification of specific types of infection risks. However, clinicians can still decide on appropriate prophylactic measures for high-risk patients based on clinical experience. For example, including antiviral drugs to prevent Herpes zoster reactivation when implementing Bortezomib-based regimens because of the potent immunosuppressive effects on T cells by Bortezomib (32, 33). Secondly, it was reported that high cumulative doses or prolonged treatment with glucocorticoids were independently associated with an increased risk of bacterial infection (24). The application of glucocorticoids was not independently analyzed in this study. Therefore, further research is required to examine this theory. Thirdly, some patients could acquire infections during treatment intervals while not being hospitalized, resulting in a low infection rate in our present study. Fourthly, though the score has been validated in an external cohort, the score is built on records from a single Center, therefore there could be a bias about infection management or prophylaxis. Last but not least, data from medical records have been retrospectively assessed, consequently, some data might be missed, and the grading might be imprecise.

In conclusion, this large cohort study confirmed that the risk of early infection in patients with MM was high. PS ≥2, HGB <35g/L of the lower limit of normal range, β2MG ≥6.0mg/L and GLB ≥2.1times the upper limit of normal range were associated with early grade ≥ 3 infection during the first 3 months. IRMM defined high-, moderate- and low-risk infection groups and showed significantly different early infection rates. Clinicians can evaluate the infection risk of patients based on the IRMM and determine appropriate and adjusted treatment strategies for various risk groups and apply prophylaxis to high-risk patients, such as vaccinations or immunoglobulin replacement or prophylactic antibiotics for the primary prevention of infection. We believe that IRMM has great potential to distinguish patients with a higher risk of infections, help to minimize adverse events, and improve outcomes of patients with MM.

## Data Availability Statement

The raw data supporting the conclusions of this article will be made available by the authors, without undue reservation.

## Ethics Statement

Ethical review and approval was not required for the study on human participants in accordance with the local legislation and institutional requirements. Written informed consent for participation was not required for this study in accordance with the national legislation and the institutional requirements.

## Author Contributions

YS designed the images and wrote the manuscript. WW, ML, and XC collected the data. YL and QW helped revise and submit the manuscript. NK modified the manuscript language. ZX and FZ designed the project, provided professional guidance and revised the manuscript. All authors contributed to the article and approved the submitted version.

## Funding

This work was supported by the National Natural Science Foundation of China (No. 81770179), and the Innovation Fund of Wuhan Optoelectronics National Lab (WNLO) (grant number 018WNLOKF023).

## Conflict of Interest

The authors declare that the research was conducted in the absence of any commercial or financial relationships that could be construed as a potential conflict of interest.

## Publisher’s Note

All claims expressed in this article are solely those of the authors and do not necessarily represent those of their affiliated organizations, or those of the publisher, the editors and the reviewers. Any product that may be evaluated in this article, or claim that may be made by its manufacturer, is not guaranteed or endorsed by the publisher.

## Supplementary Material

Supplementary Table S1 Microbiology of infections during the first 3 months and entire course.

Supplementary Table S2 Localization and microbiology of infections by treatment arm during the first 3 months. *represent Bacterial+ Herpes zoster virus.^ represent Bacterial+ Viral influenza. IMiD, immunomodulatory drugs; PI, proteasome inhibitors

Supplementary Table S3 Comparison of variables between the training cohort and validation cohort to justify the 540 cases being randomly assigned. *P* values were calculated by Mann-Whitney U test, χ² test, or Fisher’s exact test, where appropriate. ALB, Albumin; β2-MG, β2-microglobulin; BM, bone marrow; CREA, Creatinine; CRP, C reactive protein; DS, Durie-Salmon; ECOG PS, Eastern Cooperative Oncology Group performance status; GLB, Globulin; HCT, Hematocrit; HGB, Hemoglobin; ISS, International Staging System; LDH, lactate dehydrogenase; PLT, Platelet; UA, Uric acid.

Supplementary Figure S1 The possibility of infection in different risk groups for different treatment options. Time to infection in the first 3 months for high-, moderate- and low-risk groups for immunomodulatory-based regimens **(A)**, proteasome inhibitor-based regimens **(B)** and combined immunomodulatory and proteasome inhibitor **(C)** in the training and validation cohort. Time to infection in the first 3 months for high-, moderate- and low-risk groups for immunomodulatory-based regimens **(D)**, proteasome inhibitor-based regimens **(E)** and combined immunomodulatory and proteasome inhibitor **(F)** in the external validation cohort.
