# Supplementary material for: Development of a Risk Assessment Model for Early Grade ≥ 3 Infection During the First 3 Months in Patients Newly Diagnosed With Multiple Myeloma Based on a Multicenter, Real-World Analysis in China
Source: Front Oncol. 2022 Mar 17;12:772015. doi: 10.3389/fonc.2022.772015 (PMC8967980; doi:10.3389/fonc.2022.772015)
Supplement: Supplementary Table S1 — Microbiology of infections during the first 3 months and entire course. [file DataSheet_2.docx]

Table S1. Microbiology of infections during the first 3 months and entire course.

| Infectious pathogens | 0-3 Months, n | Entire course, n |
| --- | --- | --- |
| Bacterial | | |
| Streptococcal | 8 | 9 |
| Staphylococcal | 7 | 8 |
| Pseudomonal | 2 | 4 |
| Tuberculosis | 2 | 2 |
| Klebsiella | 1 | 2 |
| Escherichia | 0 | 1 |
| Baumanii | 0 | 2 |
| Multiple bacteria | 3 | 3 |
| Others | 3 | 4 |
| Fungal | | |
| Candida | 1 | 5 |
| Aspergillus | 2 | 5 |
| Blastocystis | 2 | 3 |
| Viral | | |
| Herpes | 6 | 10 |
| Influenza | 2 | 5 |
| Hepatitis | 0 | 1 |
| Bacterial and Fungal | 7 | 10 |
| Bacterial and Viral | 1 | 2 |

Table S2. Localization and microbiology of infections by treatment arm during the first 3 months.

|  | Infections during the first 3 months (110 cases) | | |
| --- | --- | --- | --- |
| Items | PI-based  (n=49) | IMiD-based  (n=12) | Combined IMiD and PI (n=19) |
| Localization |  |  |  |
| Lung and respiratory tract | 39(79.6) | 11(91.7) | 16(84.2) |
| Urinary tract | 1(2.0) | 0 | 1(5.3) |
| Skin | 2(4.1) | 1(8.3) | 2(10.5) |
| Gastrointestinal tract | 4(8.2) | 0 | 0 |
| Blood stream or bone marrow | 2(4.1) | 0 | 0 |
| Multiple sites | 1(2.0) | 0 | 0 |
| Microbiology of Infections |  |  |  |
| Bacterial (Not tuberculosis) | 15(30.6) | 0 | 4(21.1) |
| Tuberculosis | 0 | 0 | 0 |
| Fungal | 3(6.1) | 0 | 1(5.3) |
| Herpes zoster viral | 2(4.1) | 1(8.3) | 3(15.8) |
| Viral Influenza | 0 | 0 | 0 |
| Hepatitis B virus | 0 | 0 | 0 |
| Bacterial+ Fungal | 4(8.2) | 0 | 0 |
| Bacterial+ Viral | 1(2.0)* | 0 | 0 |
| Undefined pathogen | 24(49.0) | 11(91.7) | 11(57.9) |

*represent Bacterial+ Herpes zoster virus

^ represent Bacterial+ Viral influenza

IMiD, immunomodulatory drugs; PI, proteasome inhibitors

Table S3. Comparison of variables between the training cohort and validation cohort to justify the 540 cases being randomly assigned.

| Characteristics | Training cohort (n=365)  Median (IQR) or No. (%) | Validation cohort (n=175)  Median (IQR) or No. (%) | *P* |
| --- | --- | --- | --- |
| Sex/Male | 225(61.6) | 95(54.3) | 0.103 |
| Age (years) | 58.0(51.0-64.0) | 59.0(52.0-64.0) | 0.329 |
| HGB (g/L) | 100.0(76.0-120.0) | 101.0(75.0-119.6) | 0.613 |
| HCT (%) | 30.1(23.7-36.6) | 31.9(24.4-35.8) | 0.570 |
| PLT (x10^9^/L) | 194.0(143.0-266.0) | 201.0(148.0-268.0) | 0.694 |
| ALB (g/L) | 35.9(30.0-41.1) | 35.5(30.4-40.7) | 0.962 |
| GLB (g/L) | 53.4(29.9-77.2) | 49.0(29.6-80.5) | 0.800 |
| CREA (umol/L) | 81.9(62.9-115.1) | 82.3(58.4-116.9) | 0.655 |
| CRP (mg/L) | 3.4(1.1-10.8) | 3.5(0.9-10.5) | 0.920 |
| LDH (U/L) | 165.6(136.2-215.8) | 168.3(130.1-214.7) | 0.900 |
| UA (umol/L) | 424.9(329.5-546.3) | 436.0(333.2-504.7) | 0.330 |
| β2-MG (mg/L) | 4.8(3.1-7.9) | 4.4(3.1-7.2) | 0.317 |
| C3 (g/L) | 1.0(0.8-1.2) | 1.0(0.7-1.2) | 0,871 |
| C4 (g/L) | 0.2(0.1-0.3) | 0.2(0.1-0.3) | 0.798 |
| IgA (g/L) | 0.55(0.25-2.69) | 0.64(0.26-4.13) | 0.108 |
| IgG (g/L) | 18.3(5.8-56.7) | 10.4(5.5-51.9) | 0.201 |
| IgM (g/L) | 0.22(0.17-0.48) | 0.23(0.17-0.47) | 0.698 |
| Kap (g/L) | 4.7(1.3-20.6) | 4.3(1.3-15.7) | 0.636 |
| Lam (g/L) | 2.0(0.6-15.6) | 3.1(0.7-13.4) | 0.621 |
| Albumin (%) | 42.7(31.3-54.9) | 44.8(31.3-56,0) | 0.697 |
| Alpha1 (%) | 2.8(2.3-3.5) | 2.8(2.3-3.6) | 0.637 |
| Alpha2 (%) | 8.9(7.2-11.0) | 9.2(7.2-11.3) | 0.388 |
| Beta (%) | 9.4(7.4-12.1) | 9.5(7.5-12.8) | 0.794 |
| Gamma (%) | 28.1(12.9-47.9) | 26.8(11.3-47.1) | 0.389 |
| M protein (%) | 30.9(12.2-46.3) | 28.5(11.6-46.7) | 0.576 |
| Plasma cell in BM% | 15.0(6.5-32.5) | 17.0(6.5-34.5) | 0.855 |
| Bone lesions>3 | 270(74.0) | 118(67.4) | 0.114 |
| ISS III | 154(42.2) | 67(38.3) | 0.388 |
| DS IIIB | 61(16.7) | 28(16.0) | 0.835 |
| ECOG PS≥2 | 52(14.2) | 24(13.7) | 0.868 |
| Renal dysfunction | 65(17.8) | 31(17.7) | 0.979 |
| Cardiac disease | 20(5.5) | 9(5.1) | 0.871 |

*P* values were calculated by Mann-Whitney U test, χ² test, or Fisher’s exact test, where appropriate.

ALB, Albumin; β2-MG, β2-microglobulin; BM, bone marrow; CREA, Creatinine; CRP, C reactive protein; DS, Durie-Salmon; ECOG PS, Eastern Cooperative Oncology Group performance status; GLB, Globulin; HCT, Hematocrit; HGB, Hemoglobin; ISS, International Staging System; LDH, lactate dehydrogenase; PLT, Platelet; UA, Uric acid.


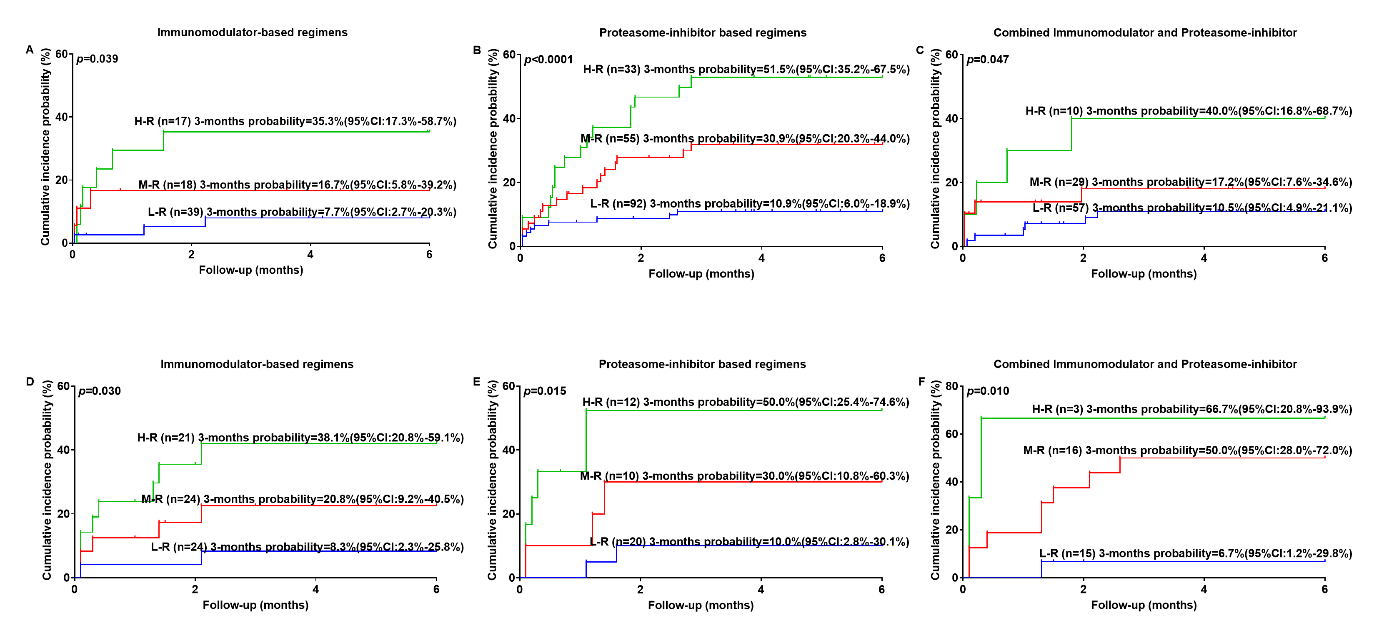


**Figure S1.** The possibility of infection in different risk groups for different treatment options. Time to infection in the first 3 months for high-, moderate- and low-risk groups for immunomodulatory-based regimens (A), proteasome inhibitor-based regimens (B) and combined immunomodulatory and proteasome inhibitor (C) in the training and validation cohort. Time to infection in the first 3 months for high-, moderate- and low-risk groups for immunomodulatory-based regimens (D), proteasome inhibitor-based regimens (E) and combined immunomodulatory and proteasome inhibitor (F) in the external validation cohort.
