# Supplementary material for: Development of a Risk Assessment Model for Early Grade ≥ 3 Infection During the First 3 Months in Patients Newly Diagnosed With Multiple Myeloma Based on a Multicenter, Real-World Analysis in China
Source: Front Oncol. 2022 Mar 17;12:772015. doi: 10.3389/fonc.2022.772015 (PMC8967980; doi:10.3389/fonc.2022.772015)
Supplement: Supplementary file 2 [file DataSheet_1.pdf]

Patient ID	Sex	Age	First Treatment	Transplantation	Infection
P1	Male	48	doxorubicin+vincristine+dexamethasone+Thalidomide	No	No
P2	Male	60	Bortezomib+Adriamycin+dexamethasone	No	Yes
P3	Male	57	Thalidomide	No	No
P4	Male	63	doxorubicin+vincristine+dexamethasone+Thalidomide	No	No
P5	Male	65	others	No	Yes
P6	Female	52	cyclophosphamide+Thalidomide+dexamethasone	No	No
P7	Male	52	doxorubicin+vincristine+dexamethasone	No	No
P8	Female	52	Isazomid+Lenalidomide+dexamethasone	Yes	No
P9	Female	70	Bortezomib+Thalidomide+dexamethasone	No	No
P10	Male	60	Bortezomib+Thalidomide+dexamethasone	No	Yes
P11	Male	44	Bortezomib+Thalidomide+dexamethasone	Yes	Yes
P12	Female	63	Bortezomib+Lenalidomide+dexamethasone	Yes	No
P13	Male	62	vincristine+dexamethasone	No	Yes
P14	Male	61	cyclophosphamide+Thalidomide+dexamethasone	No	No
P15	Male	57	Bortezomib+Ranaldomide+doxorubicin+vincristine+dexamethasone	No	Yes
P16	Male	20	doxorubicin+vincristine+dexamethasone+Thalidomide	Yes	Yes
P17	Male	68	doxorubicin+vincristine+dexamethasone	No	Yes
P18	Male	59	Thalidomide+dexamethasone	No	No
P19	Male	58	Bortezomib+cyclophosphamide+dexamethasone	No	Yes
P20	Male	58	Thalidomide	No	No
P21	Male	60	Lenalidomide+dexamethasone	No	No
P22	Male	67	doxorubicin+vincristine+dexamethasone	No	No
P23	Male	49	cyclophosphamide+Thalidomide+dexamethasone	No	No
P24	Male	52	doxorubicin+vincristine+dexamethasone+Thalidomide	No	No
P25	Female	64	doxorubicin+vincristine+dexamethasone+Thalidomide	No	No
P26	Male	62	cyclophosphamide+Thalidomide+dexamethasone	No	Yes
P27	Male	64	cyclophosphamide+Thalidomide+dexamethasone	No	Yes
P28	Male	58	Thalidomide	No	Yes
P29	Male	43	Bortezomib+Adriamycin+dexamethasone	No	Yes
P30	Male	38	Bortezomib+dexamethasone	No	Yes
P31	Male	42	doxorubicin+vincristine+dexamethasone+Thalidomide	No	Yes
P32	Male	67	doxorubicin+vincristine+dexamethasone	No	No
P33	Female	60	Bortezomib+Adriamycin+dexamethasone	No	No
P34	Female	59	Bortezomib+dexamethasone	No	Yes
P35	Male	72	Thalidomide	No	No
P36	Male	50	vincristine+cyclophosphamide+dexamethasone	No	Yes
P37	Male	60	Bortezomib+dexamethasone	No	No
P38	Male	67	Bortezomib+dexamethasone	No	No
P39	Male	43	doxorubicin+vincristine+dexamethasone	No	No
P40	Female	43	Bortezomib+dexamethasone	No	Yes
P41	Female	61	Bortezomib+Adriamycin+dexamethasone	No	Yes
P42	Female	68	cyclophosphamide+Thalidomide+dexamethasone	No	Yes
P43	Female	66	cyclophosphamide+Thalidomide+dexamethasone	No	No
P44	Male	62	doxorubicin+vincristine+dexamethasone	No	Yes
P45	Female	62	cyclophosphamide+Thalidomide+dexamethasone	No	No
P46	Male	65	cyclophosphamide+Thalidomide+dexamethasone	No	No
P47	Male	59	Bortezomib+Adriamycin+dexamethasone	No	No
P48	Female	57	Bortezomib+cyclophosphamide+dexamethasone	No	No
P49	Male	66	doxorubicin+vincristine+dexamethasone+Thalidomide	No	No
P50	Female	43	cyclophosphamide+Thalidomide+dexamethasone	No	No
P51	Male	62	doxorubicin+vincristine+dexamethasone+Thalidomide	No	No
P52	Male	45	doxorubicin+vincristine+dexamethasone	No	No
P53	Male	47	cyclophosphamide+Thalidomide+dexamethasone	No	No
P54	Male	53	Bortezomib+cyclophosphamide+dexamethasone	No	Yes
P55	Female	50	Bortezomib+Thalidomide+dexamethasone	Yes	No
P56	Female	57	Bortezomib+Thalidomide+dexamethasone	No	No
P57	Male	36	Bortezomib+Adriamycin+dexamethasone	No	No
P58	Male	46	others	No	No
P59	Male	49	Bortezomib+Thalidomide+dexamethasone	Yes	No
P60	Male	46	Bortezomib+cyclophosphamide+dexamethasone	Yes	Yes
P61	Female	63	Bortezomib+cyclophosphamide+dexamethasone	No	No
P62	Male	57	doxorubicin+vincristine+dexamethasone+Thalidomide	No	Yes
P63	Male	52	cyclophosphamide+Thalidomide+dexamethasone	No	No
P64	Female	61	cyclophosphamide+Thalidomide+dexamethasone	No	No
P65	Male	58	doxorubicin+vincristine+dexamethasone	No	Yes
P66	Male	51	doxorubicin+vincristine+dexamethasone+Thalidomide	No	Yes
P67	Female	62	Bortezomib+Thalidomide+dexamethasone	No	No
P68	Male	56	others	No	Yes
P69	Male	57	cyclophosphamide+Thalidomide+dexamethasone	No	Yes
P70	Female	52	vincristine+cyclophosphamide+dexamethasone	No	No

P71	Female	64	Bortezomib+cyclophosphamide+dexamethasone	No	No
P72	Male	42	Thalidomide	No	No
P73	Male	54	Bortezomib+dexamethasone	Yes	Yes
P74	Male	75	Bortezomib+Adriamycin+dexamethasone	No	Yes
P75	Male	56	Bortezomib+cyclophosphamide+dexamethasone	No	No
P76	Male	60	Bortezomib+Thalidomide+dexamethasone	Yes	Yes
P77	Female	47	Bortezomib+Lenalidomide+dexamethasone	No	No
P78	Male	64	Bortezomib+cyclophosphamide+dexamethasone	No	Yes
P79	Male	74	vincristine+dexamethasone	No	Yes
P80	Male	48	Bortezomib+Adriamycin+dexamethasone	Yes	No
P81	Female	49	Bortezomib+Thalidomide+dexamethasone	No	No
P82	Female	55	Bortezomib+Thalidomide+dexamethasone	No	Yes
P83	Male	66	Melphalan+prednison+	No	Yes
P84	Female	71	others	No	No
P85	Male	53	Bortezomib+Thalidomide+dexamethasone	No	No
P86	Female	49	doxorubicin+vincristine+dexamethasone	No	No
P87	Male	32	Bortezomib+Adriamycin+dexamethasone	No	Yes
P88	Male	59	cyclophosphamide+Thalidomide+dexamethasone	No	Yes
P89	Female	57	doxorubicin+vincristine+dexamethasone	No	Yes
P90	Female	51	Bortezomib+Lenalidomide+dexamethasone	No	No
P91	Male	62	Bortezomib+Adriamycin+dexamethasone	No	No
P92	Male	73	Bortezomib+Thalidomide+dexamethasone	No	No
P93	Female	49	doxorubicin+vincristine+dexamethasone	No	Yes
P94	Female	53	Bortezomib+Thalidomide+dexamethasone	No	Yes
P95	Female	39	cyclophosphamide+Thalidomide+dexamethasone	No	No
P96	Female	73	cyclophosphamide+Thalidomide+dexamethasone	No	No
P97	Male	55	others	No	Yes
P98	Female	63	doxorubicin+vincristine+dexamethasone+Thalidomide	No	Yes
P99	Male	57	doxorubicin+vincristine+dexamethasone	No	No
P100	Female	58	doxorubicin+vincristine+dexamethasone	No	No
P101	Male	59	others	No	Yes
P102	Male	68	Bortezomib+dexamethasone	No	No
P103	Male	46	Thalidomide	No	No
P104	Male	57	vincristine+vincristine+dexamethasone	No	Yes
P105	Male	74	doxorubicin+vincristine+dexamethasone	No	Yes
P106	Male	58	Thalidomide	No	Yes
P107	Female	56	Melphalan+prednison+Thalidomide	No	No
P108	Female	58	Bortezomib+Lenalidomide+dexamethasone	No	No
P109	Male	63	others	No	No
P110	Male	63	doxorubicin+vincristine+dexamethasone	No	No
P111	Male	66	doxorubicin+vincristine+dexamethasone	No	Yes
P112	Female	65	doxorubicin+vincristine+dexamethasone	No	No
P113	Female	48	doxorubicin+vincristine+dexamethasone+Thalidomide	No	No
P114	Male	55	Bortezomib+Adriamycin+dexamethasone	No	No
P115	Male	52	Bortezomib+Adriamycin+dexamethasone	No	Yes
P116	Female	46	Bortezomib+Lenalidomide+dexamethasone	Yes	No
P117	Male	56	Bortezomib+cyclophosphamide+dexamethasone	Yes	Yes
P118	Female	64	Bortezomib+Adriamycin+dexamethasone	No	Yes
P119	Female	68	doxorubicin+vincristine+dexamethasone	No	Yes
P120	Male	41	cyclophosphamide+Thalidomide+prednison	No	Yes
P121	Female	51	Bortezomib+Adriamycin+dexamethasone	No	No
P122	Male	48	cyclophosphamide+Thalidomide+dexamethasone	No	Yes
P123	Female	60	others	No	No
P124	Female	59	Bortezomib+Lenalidomide+dexamethasone	No	No
P125	Female	47	cyclophosphamide+Thalidomide+dexamethasone	No	No
P126	Male	41	doxorubicin+vincristine+dexamethasone	No	No
P127	Male	52	Bortezomib+dexamethasone	Yes	No
P128	Male	69	cyclophosphamide+Thalidomide+dexamethasone	No	No
P129	Male	76	Bortezomib+cyclophosphamide+dexamethasone	No	No
P130	Male	77	others	No	No
P131	Female	67	doxorubicin+vincristine+dexamethasone	No	No
P132	Female	54	others	No	Yes
P133	Female	64	doxorubicin+vincristine+dexamethasone	No	No
P134	Female	61	Bortezomib+cyclophosphamide+dexamethasone	No	Yes
P135	Female	59	Bortezomib+Adriamycin+dexamethasone	Yes	No
P136	Female	51	doxorubicin+vincristine+dexamethasone	No	Yes
P137	Male	52	doxorubicin+vincristine+dexamethasone	No	Yes
P138	Male	82	Bortezomib+dexamethasone	No	Yes
P139	Female	65	doxorubicin+vincristine+dexamethasone	No	Yes
P140	Male	77	others	No	No
P141	Male	76	Lenalidomide+dexamethasone	No	No

P142	Male	78	Bortezomib+cyclophosphamide+dexamethasone	No	Yes
P143	Male	44	doxorubicin+vincristine+dexamethasone	No	No
P144	Female	59	doxorubicin+vincristine+dexamethasone	No	No
P145	Male	61	doxorubicin+vincristine+dexamethasone+Thalidomide	No	No
P146	Male	84	others	No	No
P147	Female	54	Bortezomib+cyclophosphamide+dexamethasone	No	Yes
P148	Male	44	doxorubicin+vincristine+dexamethasone+Thalidomide	No	Yes
P149	Male	62	Bortezomib+Adriamycin+dexamethasone	Yes	No
P150	Male	66	doxorubicin+vincristine+dexamethasone	No	Yes
P151	Female	56	cyclophosphamide+Thalidomide+dexamethasone	No	Yes
P152	Female	66	Bortezomib+cyclophosphamide+dexamethasone	No	No
P153	Male	76	others	No	No
P154	Male	57	Bortezomib+Adriamycin+dexamethasone	No	Yes
P155	Female	38	others	No	No
P156	Female	48	doxorubicin+vincristine+dexamethasone+Thalidomide	No	Yes
P157	Female	64	others	No	No
P158	Male	61	others	No	No
P159	Female	59	others	No	No
P160	Male	72	Bortezomib+Lenalidomide+dexamethasone	No	Yes
P161	Male	34	Bortezomib+Adriamycin+dexamethasone	Yes	No
P162	Male	49	Bortezomib+Adriamycin+dexamethasone	No	No
P163	Male	64	doxorubicin+vincristine+dexamethasone	No	No
P164	Female	69	doxorubicin+vincristine+dexamethasone	No	Yes
P165	Female	47	Bortezomib+Adriamycin+dexamethasone	No	Yes
P166	Female	58	Bortezomib+Adriamycin+dexamethasone	No	Yes
P167	Female	74	Bortezomib+Adriamycin+dexamethasone	No	No
P168	Female	61	cyclophosphamide+Thalidomide+dexamethasone	No	No
P169	Male	48	others	No	Yes
P170	Male	59	doxorubicin+vincristine+dexamethasone	No	No
P171	Male	66	Bortezomib+cyclophosphamide+dexamethasone	No	Yes
P172	Female	57	doxorubicin+vincristine+dexamethasone	No	No
P173	Male	57	doxorubicin+vincristine+dexamethasone	No	No
P174	Female	63	doxorubicin+vincristine+dexamethasone	No	No
P175	Female	64	Bortezomib+Adriamycin+dexamethasone	No	Yes
P176	Male	70	others	No	Yes
P177	Female	47	doxorubicin+vincristine+dexamethasone+Thalidomide	No	Yes
P178	Male	45	Bortezomib+Thalidomide+dexamethasone	Yes	Yes
P179	Male	77	others	No	Yes
P180	Female	69	others	No	No
P181	Female	49	Bortezomib+Thalidomide+dexamethasone	Yes	No
P182	Female	61	others	No	No
P183	Female	53	Bortezomib+Adriamycin+dexamethasone	No	Yes
P184	Female	65	Bortezomib+Lenalidomide+dexamethasone	No	Yes
P185	Male	57	Bortezomib+Adriamycin+dexamethasone	No	Yes
P186	Male	60	Bortezomib+Adriamycin+dexamethasone	No	Yes
P187	Male	61	Bortezomib+Adriamycin+dexamethasone	No	No
P188	Female	67	Bortezomib+dexamethasone	No	Yes
P189	Male	45	Bortezomib+Adriamycin+dexamethasone	No	Yes
P190	Female	51	cyclophosphamide+Thalidomide+dexamethasone	No	No
P191	Male	62	Bortezomib+Adriamycin+dexamethasone	No	Yes
P192	Female	53	cyclophosphamide+Thalidomide+dexamethasone	No	No
P193	Female	37	Bortezomib+Adriamycin+dexamethasone	No	No
P194	Female	57	Bortezomib+Adriamycin+dexamethasone	No	No
P195	Female	28	doxorubicin+vincristine+dexamethasone	No	No
P196	Female	57	Bortezomib+Adriamycin+dexamethasone	No	No
P197	Female	34	Bortezomib+Adriamycin+dexamethasone	No	Yes
P198	Female	57	doxorubicin+vincristine+dexamethasone	No	No
P199	Female	62	cyclophosphamide+Thalidomide+dexamethasone	No	Yes
P200	Female	63	doxorubicin+vincristine+dexamethasone	No	No
P201	Male	75	others	No	No
P202	Female	59	Bortezomib+Lenalidomide+dexamethasone	No	No
P203	Female	63	doxorubicin+vincristine+dexamethasone	No	No
P204	Male	53	cyclophosphamide+Thalidomide+dexamethasone	No	Yes
P205	Male	62	doxorubicin+vincristine+dexamethasone	No	No
P206	Male	51	Bortezomib+Thalidomide+dexamethasone	No	Yes
P207	Female	70	Bortezomib+Adriamycin+dexamethasone	No	Yes
P208	Female	66	cyclophosphamide+Thalidomide+dexamethasone	No	No
P209	Male	85	Bortezomib+dexamethasone	No	No
P210	Male	61	others	No	No
P211	Male	42	Bortezomib+Adriamycin+dexamethasone	No	No
P212	Female	52	Bortezomib+Adriamycin+dexamethasone	No	No

P213	Female	68	doxorubicin+vincristine+dexamethasone	No	No
P214	Male	51	Bortezomib+Adriamycin+dexamethasone	No	No
P215	Male	56	Bortezomib+Adriamycin+dexamethasone	No	Yes
P216	Female	64	others	No	No
P217	Male	57	Bortezomib+Adriamycin+dexamethasone	No	No
P218	Male	57	Bortezomib+Adriamycin+dexamethasone	No	No
P219	Male	47	Thalidomide+dexamethasone	No	No
P220	Male	52	cyclophosphamide+Thalidomide+dexamethasone	No	Yes
P221	Female	59	doxorubicin+vincristine+dexamethasone	No	Yes
P222	Male	67	Bortezomib+Thalidomide+dexamethasone	No	No
P223	Male	59	others	No	No
P224	Male	62	doxorubicin+vincristine+dexamethasone	No	No
P225	Female	43	doxorubicin+vincristine+dexamethasone	No	No
P226	Male	61	others	No	No
P227	Female	63	others	No	No
P228	Male	64	others	No	No
P229	Male	43	Bortezomib+Adriamycin+dexamethasone	No	No
P230	Male	62	Bortezomib+Adriamycin+dexamethasone	No	Yes
P231	Female	30	others	No	No
P232	Male	59	Bortezomib+Lenalidomide+dexamethasone	Yes	No
P233	Male	53	Bortezomib+Lenalidomide+dexamethasone	Yes	Yes
P234	Male	72	Bortezomib+cyclophosphamide+dexamethasone	No	Yes
P235	Male	54	others	No	No
P236	Male	55	others	No	No
P237	Male	63	Bortezomib+cyclophosphamide+dexamethasone	Yes	No
P238	Female	66	Bortezomib+Adriamycin+dexamethasone	No	No
P239	Female	51	Bortezomib+cyclophosphamide+dexamethasone	No	No
P240	Male	51	Bortezomib+Lenalidomide+dexamethasone	Yes	No
P241	Male	26	Bortezomib+Adriamycin+dexamethasone	Yes	No
P242	Female	59	Bortezomib+Lenalidomide+dexamethasone	Yes	No
P243	Female	62	Bortezomib+Lenalidomide+dexamethasone	No	No
P244	Male	49	Bortezomib+Lenalidomide+dexamethasone	Yes	No
P245	Female	36	Bortezomib+Adriamycin+dexamethasone	No	No
P246	Male	69	Bortezomib+cyclophosphamide+dexamethasone	No	No
P247	Male	65	others	No	No
P248	Female	61	others	No	No
P249	Male	40	others	No	No
P250	Female	52	Bortezomib+Adriamycin+dexamethasone	No	Yes
P251	Male	47	Bortezomib+Adriamycin+dexamethasone	Yes	Yes
P252	Female	52	Bortezomib+dexamethasone	No	No
P253	Female	65	Bortezomib+Adriamycin+dexamethasone	No	No
P254	Male	56	Bortezomib+cyclophosphamide+dexamethasone	No	No
P255	Male	53	Bortezomib+Adriamycin+dexamethasone	No	Yes
P256	Female	51	others	No	No
P257	Male	64	Bortezomib+Adriamycin+dexamethasone	No	No
P258	Female	51	Bortezomib+Lenalidomide+dexamethasone	No	No
P259	Male	55	Bortezomib+Adriamycin+dexamethasone	No	No
P260	Female	64	Bortezomib+Thalidomide+dexamethasone	Yes	No
P261	Male	54	Bortezomib+Adriamycin+dexamethasone	No	Yes
P262	Male	36	Bortezomib+Lenalidomide+dexamethasone	Yes	Yes
P263	Female	70	others	No	No
P264	Male	51	Bortezomib+cyclophosphamide+dexamethasone	Yes	No
P265	Female	55	Bortezomib+Adriamycin+dexamethasone	No	No
P266	Female	63	Bortezomib+Lenalidomide+dexamethasone	No	No
P267	Male	74	Lenalidomide+dexamethasone	No	No
P268	Female	56	Bortezomib+Adriamycin+dexamethasone	No	No
P269	Male	59	Bortezomib+Adriamycin+dexamethasone	No	No
P270	Male	60	Bortezomib+Adriamycin+dexamethasone	No	Yes
P271	Male	51	Bortezomib+Adriamycin+dexamethasone	No	No
P272	Male	51	Bortezomib+Lenalidomide+dexamethasone	Yes	Yes
P273	Female	44	Bortezomib+Lenalidomide+dexamethasone	Yes	No
P274	Male	68	others	No	No
P275	Female	28	others	No	No
P276	Female	55	others	No	No
P277	Female	62	Bortezomib+Thalidomide+dexamethasone	No	No
P278	Female	52	Bortezomib+cyclophosphamide+dexamethasone	No	No
P279	Male	60	Bortezomib+Adriamycin+dexamethasone	No	Yes
P280	Male	58	Bortezomib+Adriamycin+dexamethasone	No	No
P281	Male	68	Bortezomib+Adriamycin+dexamethasone	No	No

P282	Male	68	Bortezomib+Lenalidomide+dexamethasone	No	Yes
P283	Female	63	Bortezomib+Lenalidomide+dexamethasone	Yes	No
P284	Female	63	Bortezomib+Lenalidomide+dexamethasone	No	No
P285	Male	61	Bortezomib+Lenalidomide+dexamethasone	No	No
P286	Female	64	Bortezomib+Adriamycin+dexamethasone	No	Yes
P287	Male	50	cyclophosphamide+Thalidomide+dexamethasone	No	No
P288	Female	63	Bortezomib+Adriamycin+dexamethasone	No	No
P289	Male	52	Bortezomib+Adriamycin+dexamethasone	No	No
P290	Male	75	Bortezomib+Adriamycin+dexamethasone	No	No
P291	Male	64	Bortezomib+Adriamycin+dexamethasone	No	No
P292	Male	66	Bortezomib+Adriamycin+dexamethasone	No	No
P293	Male	43	Bortezomib+Adriamycin+dexamethasone	Yes	No
P294	Male	58	others	No	No
P295	Female	64	Bortezomib+Thalidomide+dexamethasone	No	Yes
P296	Male	59	others	No	No
P297	Male	55	Bortezomib+Adriamycin+dexamethasone	No	No
P298	Male	55	Bortezomib+Adriamycin+dexamethasone	No	No
P299	Male	48	cyclophosphamide+Thalidomide+dexamethasone	No	No
P300	Male	57	Bortezomib+Lenalidomide+dexamethasone	No	No
P301	Female	64	others	No	No
P302	Male	64	others	No	No
P303	Female	78	others	No	No
P304	Male	65	Bortezomib+Adriamycin+dexamethasone	No	No
P305	Male	75	others	No	Yes
P306	Female	84	Isazomid+Lenalidomide+dexamethasone	No	No
P307	Male	52	Bortezomib+Adriamycin+dexamethasone	No	No
P308	Male	60	Bortezomib+Lenalidomide+dexamethasone	No	No
P309	Female	59	Bortezomib+Lenalidomide+dexamethasone	No	No
P310	Male	45	Bortezomib+Adriamycin+dexamethasone	No	No
P311	Male	65	Bortezomib+Thalidomide+dexamethasone	No	No
P312	Female	47	Bortezomib+Lenalidomide+dexamethasone	Yes	Yes
P313	Male	28	Bortezomib+Lenalidomide+dexamethasone	Yes	Yes
P314	Female	34	Bortezomib+Lenalidomide+dexamethasone	Yes	No
P315	Female	46	others	No	No
P316	Female	57	Bortezomib+Lenalidomide+dexamethasone	No	No
P317	Female	61	Bortezomib+Adriamycin+dexamethasone	No	Yes
P318	Male	65	Bortezomib+cyclophosphamide+dexamethasone	No	No
P319	Male	69	Bortezomib+Lenalidomide+dexamethasone	No	Yes
P320	Female	51	Bortezomib+Thalidomide+dexamethasone	No	No
P321	Female	67	others	No	No
P322	Female	70	Bortezomib+Lenalidomide+dexamethasone	No	No
P323	Male	52	others	No	No
P324	Male	58	Bortezomib+Lenalidomide+dexamethasone	No	Yes
P325	Male	69	Bortezomib+cyclophosphamide+dexamethasone	No	No
P326	Male	52	others	No	No
P327	Male	55	Bortezomib+Adriamycin+dexamethasone	No	No
P328	Male	68	Bortezomib+Lenalidomide+dexamethasone	No	No
P329	Male	76	Bortezomib+dexamethasone	No	Yes
P330	Male	70	others	No	No
P331	Female	50	Bortezomib+Adriamycin+dexamethasone	No	No
P332	Male	50	Bortezomib+Adriamycin+dexamethasone	No	Yes
P333	Male	72	Bortezomib+Lenalidomide+dexamethasone	No	Yes
P334	Male	53	others	No	No
P335	Male	74	Bortezomib+cyclophosphamide+dexamethasone	No	No
P336	Male	55	Bortezomib+Adriamycin+dexamethasone	No	No
P337	Male	46	Isazomid+dexamethasone	No	No
P338	Male	66	others	No	No
P339	Female	50	Bortezomib+Adriamycin+dexamethasone	No	No
P340	Male	71	Bortezomib+Adriamycin+dexamethasone	No	No
P341	Female	56	Bortezomib+Adriamycin+dexamethasone	No	No
P342	Female	43	Bortezomib+cyclophosphamide+dexamethasone	No	No
P343	Female	54	Bortezomib+Lenalidomide+dexamethasone	Yes	No
P344	Female	69	Bortezomib+Adriamycin+dexamethasone	No	Yes
P345	Female	48	others	No	No
P346	Male	65	Bortezomib+Adriamycin+dexamethasone	No	No
P347	Male	59	Bortezomib+Thalidomide+dexamethasone	No	No
P348	Male	62	Bortezomib+Adriamycin+dexamethasone	No	Yes
P349	Female	42	Bortezomib+Lenalidomide+dexamethasone	No	No

P350	Female	62	Isazomid+Lenalidomide+dexamethasone	No	No
P351	Male	73	Bortezomib+cyclophosphamide+dexamethasone	No	No
P352	Male	51	others	No	No
P353	Male	53	Ixazomib+Adriamycin+dexamethasone	Yes	Yes
P354	Female	57	Bortezomib+Adriamycin+dexamethasone	No	No
P355	Male	39	Isazomid+Lenalidomide+dexamethasone	Yes	Yes
P356	Female	60	Bortezomib+cyclophosphamide+dexamethasone	No	No
P357	Male	24	Bortezomib+Adriamycin+dexamethasone	No	No
P358	Female	44	Bortezomib+Adriamycin+dexamethasone	No	No
P359	Male	54	Bortezomib+Adriamycin+dexamethasone	No	No
P360	Male	50	Bortezomib+Lenalidomide+dexamethasone	Yes	No
P361	Female	58	Bortezomib+Adriamycin+dexamethasone	No	Yes
P362	Male	56	Bortezomib+Adriamycin+dexamethasone	Yes	No
P363	Male	47	cyclophosphamide+Thalidomide+dexamethasone	No	No
P364	Male	71	Bortezomib+Thalidomide+dexamethasone	No	No
P365	Male	76	Isazomid+Lenalidomide+dexamethasone	No	No
P366	Male	80	others	No	Yes
P367	Female	66	Bortezomib+Lenalidomide+dexamethasone	No	Yes
P368	Male	65	Bortezomib+Lenalidomide+dexamethasone	Yes	Yes
P369	Male	68	Bortezomib+Lenalidomide+dexamethasone	No	No
P370	Male	45	Bortezomib+Adriamycin+dexamethasone	No	No
P371	Male	58	others	No	Yes
P372	Female	68	Bortezomib+Adriamycin+dexamethasone	No	No
P373	Male	40	Bortezomib+Lenalidomide+dexamethasone	No	No
P374	Male	62	Bortezomib+Lenalidomide+dexamethasone	No	No
P375	Female	44	Isazomid+Adriamycin+dexamethasone	No	No
P376	Female	51	others	No	No
P377	Male	70	Isazomid+Lenalidomide+dexamethasone	No	Yes
P378	Male	64	Bortezomib+Adriamycin+dexamethasone	No	No
P379	Female	56	Bortezomib+Adriamycin+dexamethasone	No	No
P380	Male	56	Bortezomib+Adriamycin+dexamethasone	No	Yes
P381	Male	45	Bortezomib+Adriamycin+dexamethasone	No	Yes
P382	Female	61	Bortezomib+Adriamycin+dexamethasone	No	No
P383	Female	51	cyclophosphamide+Thalidomide+dexamethasone	No	No
P384	Male	57	Bortezomib+Adriamycin+dexamethasone	No	Yes
P385	Female	56	Bortezomib+Adriamycin+dexamethasone	No	No
P386	Male	55	Bortezomib+cyclophosphamide+dexamethasone	No	No
P387	Male	53	others	No	No
P388	Male	65	Bortezomib+Lenalidomide+dexamethasone	No	No
P389	Female	60	others	No	No
P390	Female	53	Isazomid+Lenalidomide+dexamethasone	No	No
P391	Male	72	Isazomid+Lenalidomide+dexamethasone	No	Yes
P392	Female	54	Bortezomib+Adriamycin+dexamethasone	No	No
P393	Male	73	others	No	No
P394	Male	64	Bortezomib+Lenalidomide+dexamethasone	No	No
P395	Male	63	others	No	No
P396	Female	71	others	No	No
P397	Male	52	others	No	No
P398	Female	38	Bortezomib+Adriamycin+dexamethasone	No	No
P399	Male	70	others	No	No
P400	Male	49	others	No	No
P401	Female	47	Bortezomib+Adriamycin+dexamethasone	No	No
P402	Male	46	Bortezomib+Lenalidomide+dexamethasone	Yes	Yes
P403	Male	56	Bortezomib+Adriamycin+dexamethasone	No	No
P404	Male	38	Bortezomib+Adriamycin+dexamethasone	No	No
P405	Female	46	others	No	No
P406	Female	75	Bortezomib+Lenalidomide+dexamethasone	No	No
P407	Male	57	others	No	No
P408	Female	44	Bortezomib+Lenalidomide+dexamethasone	No	No
P409	Male	48	Bortezomib+Adriamycin+dexamethasone	No	No
P410	Male	62	Bortezomib+Adriamycin+dexamethasone	No	No
P411	Female	61	Bortezomib+Adriamycin+dexamethasone	No	No
P412	Female	61	Bortezomib+dexamethasone	No	No
P413	Female	47	Bortezomib+Lenalidomide+dexamethasone	No	No
P414	Female	46	Bortezomib+Adriamycin+dexamethasone	No	No
P415	Male	76	Isazomid+Lenalidomide+dexamethasone	No	Yes
P416	Female	53	Bortezomib+Adriamycin+dexamethasone	No	Yes
P417	Female	84	Bortezomib+dexamethasone	No	No

P418	Female	46	Bortezomib+Thalidomide+dexamethasone	Yes	No
P419	Male	62	Bortezomib+dexamethasone	No	No
P420	Female	64	Bortezomib+Adriamycin+dexamethasone	No	No
P421	Male	61	Melphalan+prednison+Thalidomide	No	No
P422	Male	59	Bortezomib+Adriamycin+dexamethasone	No	Yes
P423	Male	72	Melphalan+prednison+	No	Yes
P424	Male	62	Bortezomib+Adriamycin+dexamethasone	No	No
P425	Female	61	Melphalan+prednison+Thalidomide	No	No
P426	Female	65	Bortezomib+Adriamycin+dexamethasone	No	No
P427	Female	66	Bortezomib+Adriamycin+dexamethasone	No	No
P428	Female	74	Bortezomib+Adriamycin+dexamethasone	No	No
P429	Male	51	Bortezomib+Adriamycin+dexamethasone	No	No
P430	Female	55	Bortezomib+Adriamycin+dexamethasone	No	No
P431	Male	64	Bortezomib+Adriamycin+dexamethasone	No	Yes
P432	Male	77	Melphalan+prednison+Thalidomide	No	No
P433	Female	56	Bortezomib+Adriamycin+dexamethasone	No	No
P434	Male	58	Bortezomib+dexamethasone	No	Yes
P435	Male	45	Bortezomib+Adriamycin+dexamethasone	No	No
P436	Male	62	Bortezomib+Adriamycin+dexamethasone	No	Yes
P437	Female	57	Bortezomib+Adriamycin+dexamethasone	No	No
P438	Male	56	Bortezomib+Adriamycin+dexamethasone	No	No
P439	Male	31	Melphalan+prednison+Thalidomide	No	No
P440	Female	71	Bortezomib+dexamethasone	No	No
P441	Female	63	Bortezomib+Adriamycin+dexamethasone	No	Yes
P442	Male	70	Bortezomib+dexamethasone	No	No
P443	Female	56	Bortezomib+Adriamycin+dexamethasone	No	No
P444	Male	55	Melphalan+prednison+Thalidomide	No	No
P445	Female	49	Melphalan+prednison+Thalidomide	No	No
P446	Female	68	Bortezomib+Adriamycin+dexamethasone	No	No
P447	Male	65	others	No	No
P448	Male	59	Bortezomib+Adriamycin+dexamethasone	No	No
P449	Male	57	Bortezomib+Adriamycin+dexamethasone	No	No
P450	Male	59	others	No	No
P451	Female	64	vincristine+Thalidomide+dexamethasone	No	No
P452	Male	61	Bortezomib+Thalidomide+dexamethasone	No	Yes
P453	Female	65	Bortezomib+Thalidomide+dexamethasone	Yes	No
P454	Female	51	Bortezomib+Adriamycin+dexamethasone	No	No
P455	Male	33	Bortezomib+Thalidomide+dexamethasone	No	No
P456	Female	61	Bortezomib+Thalidomide+dexamethasone	No	No
P457	Male	54	Melphalan+prednison+Thalidomide	No	No
P458	Male	70	Melphalan+prednison+Thalidomide	No	No
P459	Female	54	Bortezomib+Adriamycin+dexamethasone	No	No
P460	Male	53	Bortezomib+Lenalidomide+dexamethasone	No	Yes
P461	Female	58	Bortezomib+Thalidomide+dexamethasone	No	No
P462	Male	59	Bortezomib+Adriamycin+dexamethasone	No	No
P463	Male	71	Bortezomib+Adriamycin+dexamethasone	No	No
P464	Female	67	Bortezomib+dexamethasone	No	No
P465	Female	62	Bortezomib+Thalidomide+dexamethasone	No	Yes
P466	Male	68	Bortezomib+Thalidomide+dexamethasone	No	No
P467	Female	61	Bortezomib+Thalidomide+dexamethasone	No	No
P468	Male	57	Bortezomib+Adriamycin+dexamethasone	No	No
P469	Female	54	Melphalan+prednison+Thalidomide	No	Yes
P470	Female	56	doxorubicin+vincristine+dexamethasone	No	No
P471	Female	68	Bortezomib+Adriamycin+dexamethasone	No	Yes
P472	Male	36	Bortezomib+Thalidomide+dexamethasone	No	No
P473	Male	55	Bortezomib+Adriamycin+dexamethasone	No	No
P474	Female	61	Bortezomib+Adriamycin+dexamethasone	No	No
P475	Female	63	Bortezomib+Adriamycin+dexamethasone	No	Yes
P476	Female	65	Melphalan+prednison+Thalidomide	No	No
P477	Female	47	Bortezomib+Adriamycin+dexamethasone	No	Yes
P478	Male	58	Bortezomib+Adriamycin+dexamethasone	No	No
P479	Female	64	doxorubicin+vincristine+dexamethasone	No	No
P480	Male	61	Bortezomib+Thalidomide+dexamethasone	No	No
P481	Male	33	Bortezomib+Thalidomide+dexamethasone	No	Yes
P482	Male	63	Bortezomib+Adriamycin+dexamethasone	No	No
P483	Male	65	Bortezomib+Adriamycin+dexamethasone	No	No
P484	Male	54	Bortezomib+Adriamycin+dexamethasone	No	No
P485	Male	47	Bortezomib+Adriamycin+dexamethasone	No	No
P486	Female	46	Bortezomib+Thalidomide+dexamethasone	No	No
P487	Male	56	Bortezomib+Thalidomide+dexamethasone	No	No
P488	Female	47	Bortezomib+Thalidomide+dexamethasone	No	No

P489	Female	39	doxorubicin+vincristine+dexamethasone	No	No
P490	Female	60	doxorubicin+vincristine+dexamethasone	No	Yes
P491	Male	68	Bortezomib+Thalidomide+dexamethasone	No	No
P492	Female	64	Bortezomib+Adriamycin+dexamethasone	No	Yes
P493	Male	65	Bortezomib+Thalidomide+dexamethasone	No	Yes
P494	Male	39	doxorubicin+vincristine+dexamethasone+Thalidomide	No	No
P495	Male	45	Bortezomib+Adriamycin+dexamethasone	No	No
P496	Male	50	doxorubicin+vincristine+dexamethasone	No	No
P497	Female	54	Bortezomib+Adriamycin+dexamethasone	No	No
P498	Female	47	Bortezomib+Thalidomide+dexamethasone	Yes	Yes
P499	Female	61	Bortezomib+dexamethasone	No	Yes
P500	Male	56	doxorubicin+vincristine+dexamethasone	No	No
P501	Male	67	Melphalan+prednison+Thalidomide	No	Yes
P502	Male	63	Melphalan+prednison	No	Yes
P503	Female	61	Bortezomib+Thalidomide+dexamethasone	No	Yes
P504	Female	69	doxorubicin+vincristine+dexamethasone	No	Yes
P505	Male	52	doxorubicin+vincristine+dexamethasone	No	No
P506	Male	57	Bortezomib+Thalidomide+dexamethasone	No	Yes
P507	Male	50	doxorubicin+vincristine+dexamethasone	No	No
P508	Male	74	doxorubicin+vincristine+dexamethasone	No	No
P509	Male	56	doxorubicin+vincristine+dexamethasone	No	No
P510	Female	53	Bortezomib+Thalidomide+dexamethasone	Yes	No
P511	Male	52	Bortezomib+Thalidomide+dexamethasone	Yes	No
P512	Female	58	doxorubicin+vincristine+dexamethasone	No	Yes
P513	Female	66	doxorubicin+vincristine+dexamethasone	No	No
P514	Male	47	Bortezomib+Thalidomide+dexamethasone	Yes	No
P515	Male	68	doxorubicin+vincristine+dexamethasone	No	Yes
P516	Male	40	doxorubicin+vincristine+dexamethasone	No	No
P517	Female	47	doxorubicin+vincristine+dexamethasone+Thalidomide	No	Yes
P518	Male	63	Bortezomib+cyclophosphamide+dexamethasone	No	Yes
P519	Male	59	Bortezomib+Adriamycin+dexamethasone	No	No
P520	Female	51	Bortezomib+Adriamycin+dexamethasone	No	No
P521	Male	55	Bortezomib+Adriamycin+dexamethasone	No	No
P522	Male	71	doxorubicin+vincristine+dexamethasone	No	No
P523	Female	68	doxorubicin+vincristine+dexamethasone	No	Yes
P524	Female	62	doxorubicin+vincristine+dexamethasone	No	Yes
P525	Male	76	doxorubicin+vincristine+dexamethasone	No	No
P526	Male	50	doxorubicin+vincristine+dexamethasone	No	No
P527	Male	64	doxorubicin+vincristine+dexamethasone	No	Yes
P528	Male	72	doxorubicin+vincristine+dexamethasone	No	No
P529	Male	66	doxorubicin+vincristine+dexamethasone	No	No
P530	Female	62	Bortezomib+Adriamycin+dexamethasone	No	Yes
P531	Male	44	doxorubicin+vincristine+dexamethasone	No	No
P532	Male	45	doxorubicin+vincristine+dexamethasone	No	No
P533	Male	65	Bortezomib+dexamethasone	No	No
P534	Male	64	doxorubicin+vincristine+dexamethasone	No	Yes
P535	Male	62	doxorubicin+vincristine+dexamethasone	No	No
P536	Male	59	Bortezomib+Lenalidomide+dexamethasone	Yes	No
P537	Male	71	doxorubicin+vincristine+dexamethasone	No	No
P538	Male	80	Bortezomib+dexamethasone	No	No
P539	Male	65	doxorubicin+vincristine+dexamethasone	No	No
P540	Female	24	others	No	No
